# Coupling Mass Spectral and Genomic Information to Improve Bacterial Natural Product Discovery Workflows

**DOI:** 10.3390/md19030142

**Published:** 2021-03-05

**Authors:** Max Crüsemann

**Affiliations:** Institute for Pharmaceutical Biology, University of Bonn, Nussallee 6, 53115 Bonn, Germany; cruesemann@uni-bonn.de

**Keywords:** bacterial natural products, mass spectrometry, genome mining, paired omics

## Abstract

Bacterial natural products possess potent bioactivities and high structural diversity and are typically encoded in biosynthetic gene clusters. Traditional natural product discovery approaches rely on UV- and bioassay-guided fractionation and are limited in terms of dereplication. Recent advances in mass spectrometry, sequencing and bioinformatics have led to large-scale accumulation of genomic and mass spectral data that is increasingly used for signature-based or correlation-based mass spectrometry genome mining approaches that enable rapid linking of metabolomic and genomic information to accelerate and rationalize natural product discovery. In this mini-review, these approaches are presented, and discovery examples provided. Finally, future opportunities and challenges for paired omics-based natural products discovery workflows are discussed.

## 1. Introduction

Due to their impressive structural diversity and their wide range of bioactivities, natural products (NP) have been, and are still extensively used by humankind as important sources for drugs [1]. NP structures are generated by the concerted action of biosynthetic enzymes. These are encoded in genes which are, in bacteria, usually grouped to biosynthetic gene clusters (BGCs). This circumstance has facilitated bioinformatics analyses and predictions about the number and classes of natural products that can be synthesized by a bacterial strain [2]. This procedure is termed “genome mining”, a rapidly growing field that has advanced NP research in the last 15 years [3,4]. Sets of closely related BGCs with similar gene content can be grouped into gene cluster families (GCF), that encode the production of identical or highly similar molecules. The recent advances in DNA sequencing have led to a massive accumulation of sequence data in the databases which, in turn, fueled the development of large-scale BGC and GCF analysis pipelines and databases such as BiG-SCAPE [5], BiG-SLICE [6] and BiG-FAM [7] by Medema and colleagues. These frameworks enable the systematic estimation and comparison of NP biosynthetic potential in increasing numbers of bacterial strains.

Biosynthetic machineries usually do not only lead to one single natural product, but may produce a suite of structurally related metabolites through relaxed substrate specificities, causing enzymatic processing of structurally different precursors and intermediates. Mass spectrometry (MS)-based workflows offer opportunities to chart the metabolic diversity that is present in a complex sample, e.g., a crude bacterial extract. The metabolic diversity in complex NP mixtures can be regarded as a collection of “molecular families”, a term for structurally related compounds with related MS fragmentation (MS/MS) spectra [8]. As an outstanding example, the public community data repository and analysis platform GNPS [9,10], developed by Dorrestein, Bandeira and colleagues, offers opportunities for the detailed analysis and visualization of natural product MS/MS fragmentation data by molecular networking. The GNPS environment has also integrated several useful annotation, classification and dereplication tools [11,12,13,14,15,16,17] that, if used altogether, aid in obtaining the maximum amount of information from an MS/MS spectrum or dataset of interest.

One of the most important goals in natural product discovery and the basis for any state-of-the-art biosynthetic study is the direct linkage of a metabolite to its BGC. The classical and most reliable way to establish such a link is either the heterologous expression or the activation of a cryptic BGC, with subsequent detection and characterization of the target compounds in the heterologous or engineered host, or the deletion of the BGC or key biosynthetic genes thereof in the NP producer to abolish production of the natural product of interest. However, although significant advances have been made in these areas [18,19], these approaches are still relatively laborious and time consuming because only a single biosynthetic pathway can be targeted in one experimental workflow, that typically requires several, sometimes cumbersome cloning and transformation procedures. In contrast, MS-guided genome mining techniques that have been developed, enable the parallel establishment of multiple compound-BGC linkages and dereplication in a much more time-effective workflow. The acquisition and in-depth analysis of paired datasets comprising MS/MS data of culture extracts and genome sequences of their producers is thus of promise to accelerate and improve any bacterial NP discovery program.

Concepts and successful examples of linking chemical and biosynthetic space have lately been reviewed by Duncan and coworkers [20]. Another detailed application-oriented review by van der Hooft, Medema and colleagues mainly focused on the key technologies that enable making these linkages [21]. This minireview particularly intends to highlight concepts to directly link mass spectral information and BGCs by (i) signature-based approaches (peptidogenomics and glycogenomics), as well as (ii) correlation-based approaches (pattern-based genome mining, metabologenomics) and provides discovery examples. Finally, latest developments and future promises and challenges in linking biosynthetic and metabolomic data of natural products are presented and discussed.

## 2. Concepts and Examples for Linking Genomic and Metabolomic Data

### 2.1. Experiment-Guided Genome Mining: Peptidogenomics and Glycogenomics 

**Figure 1:**
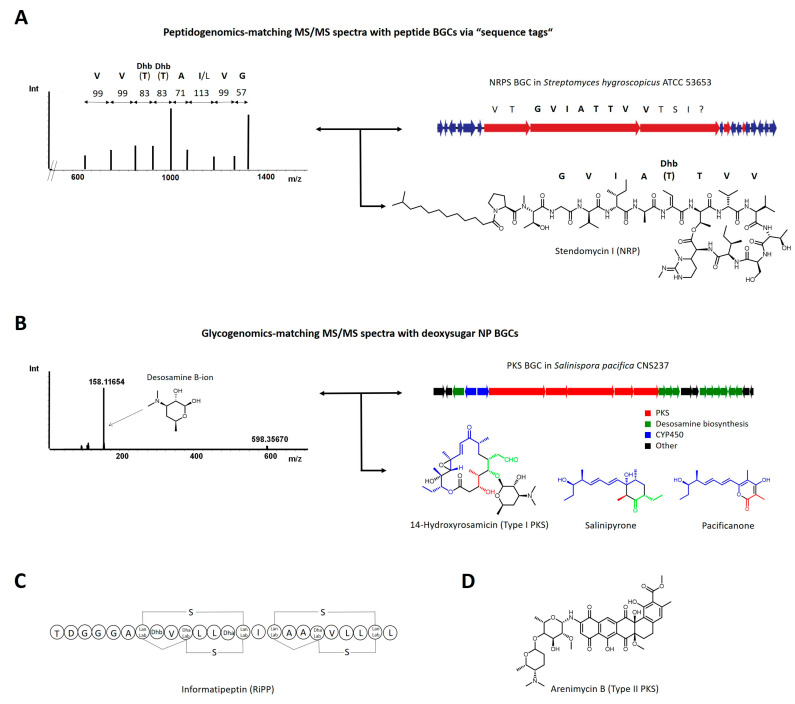
**Concepts and discovery examples for experiment-guided genome mining.** (**A**) Peptidogenomics: *Streptomyces hygroscopicus* MS/MS data yielded a sequence tag with eight amino acids, two of them dehydrated threonines (Dhb). The sequence tag matched with a sequence of adenylation domain predictions of an orphan nonribosomal peptide synthetase BGC, facilitating the targeted isolation and characterization of stendomycin lipopeptides [22]. The second threonine dehydration appeared only during MS/MS fragmentation as elimination product of the ester bond. (**B**) Glycogenomics: Matching of MS/MS spectra of *Salinispora pacifica* CNS237 with a type I polyketide synthase BGC encoding deoxysugar biosynthesis genes revealed several rosamicin derivatives and enabled their targeted isolation and further characterization. The previously isolated linear polyketides salinipyrone and pacificanone appear to be shunt products of the rosamicin PKS, revealed by mutagenesis experiments. Building blocks synthesized by the same module(s) are color-coded accordingly [33]. (**C**) Further natural products from different classes discovered by the peptidogenomic [28] and (**D**) glycogenomic approach [23]. For more details regarding these concepts, please refer to references [22,23].

These two MS-guided genome mining approaches were developed and pioneered by Kersten, Dorrestein and Moore [22,23]. Both workflows are dependent on the presence of specific signatures, i.e., mass shifts or fragmentation ions, in an MS/MS spectrum from bacterial compound mixtures. These distinctive signatures may be linked to a BGC predicted to encode the biosynthetic machinery to produce NPs with structural motifs to yield these MS/MS fragments. This procedure is particularly applicable for peptides and glycosylated molecules (Figure 1). 

In peptidogenomics, the mass shifts relate to the fragmentation of peptides into their constituents, i.e., proteinogenic or modified, non-proteinogenic amino acids, a process that allows for automation [22,24]. A number of subsequent amino acid MS/MS mass shifts constitutes a “sequence tag”, which is instrumental in the search for the respective BGC in the producers’ genome (Figure 1A). For ribosomally synthesized and modified peptide natural products (RiPP), the sequence tag is part of a small, encoded protein, usually clustered with genes encoding posttranslationally modifying enzymes, that is queried in the producers’ genome e.g., by six-frame translations. Nonribosomal peptides (NRP) are synthesized by multimodular assembly line megaenzymes. Here, a detected sequence tag relates to a BGC encoding a sequence of modules with predictable adenylation domain specificities. In glycogenomics, diagnostic mass shifts or fragments are caused by the fragmentation of bonds to sugars or, preferably, modified deoxysugars, both frequently observed features of bioactive natural products [23]. The biosynthesis of deoxysugars is typically encoded by subclusters of modifying biosynthetic genes and glycosyltransferases, clustered with genes encoding core NP biosynthetic machineries (e.g., polyketide synthases) and can be matched with the detected MS/MS deoxysugar fragment(s) (Figure 1B). 

In a landmark study in 2011, the concept of peptidogenomics was introduced to the NP community and systematically used to uncover and characterize a series of novel RiPPs from well investigated *Streptomyces* strains such as *S. lividans*, *S. coelicolor* and *S. griseus*. Additionally, five novel analogs of the nonribosomal lipopeptide stendomycin were characterized from *S. hygroscopicus* and connected to their BGC (Figure 1A) [22]. In a subsequent study, *Streptomyces roseosporus* natural products were mapped with a combination of molecular networking and peptidogenomics which led to the discovery of the stenothricin BGC [25]. Another peptidogenomic study on *S. roseosporus* based on imaging MS revealed that the potent antibiotic peptide arylomycin was of nonribosomal origin [26]. Bromoalterochromides were discovered and connected to their BGC in two marine *Pseudoalteromonas* bacteria from a large scale nano-DESI MS/MS dataset of *Bacillus* and *Pseudoalteromonas* strains [8]. From the plant pathogen *Ralstonia solanacearum*, the bioactive lipopeptide ralsolamycin was also identified using the peptidogenomic approach [27]. 

The peptidogenomic concept was automated by Pevzner, Mohimani and colleagues, leading to the development of automated peptidogenomics tools specifically designed for RiPPs (RiPP-Quest) [28], NRPs (NRP-Quest) [29] and both (Pep2Path) [30]. Application of RiPP-Quest enabled the discovery of informatipeptin (Figure 1C), a new class III lanthipeptide from *Streptomyces viridochromogenes*. Recently, the development of MetaMiner, an advancement of the RiPP-Quest tool, designed for the query of larger datasets e.g., from metagenomes, and its application to several datasets in the GNPS database led to the discovery and annotation of seven previously unknown RiPPs [31].

The development of the glycogenomic approach and its proof-of-principle application to a set of marine actinomycete crude extracts enabled discovery and MS-guided isolation of arenimycin B (Figure 1D), a type II polyketide comprising the two characteristic deoxysugar moieties forosamine and O-methyl rhamnose from the marine actinomycete *Salinispora arenicola* CNB527 [23]. The derivative arenimycin A, containing only O-methyl rhamnose, had previously been isolated from another *S. arenicola* strain [32] by classical, UV-guided purification. However, analysis of the biosynthetic potential of CNB527 suggested that a candidate BGC for arenimycin biosynthetic machinery could be capable of adding another sugar moiety to the molecule. After its characterization, it was thus concluded that arenimycin B is actually the end product of the biosynthetic pathway, and was notably found to be more bioactive than the previously isolated arenimycin A, showing a twofold or greater increase in activity against clinically relevant, multidrug-resistant strains of *Staphylococcus aureus* [23]. Another glycogenomic example with marine origin is the discovery of five rosamicin derivatives from the marine actinomycete *Salinispora pacifica* CNS237 (Figure 1B) [33]. This group of antibiotically active, glycosylated polyketides, among them three unprecedented analogs, was discovered by their characteristic desosamine fragment from a large MS/MS dataset that was used to prioritize marine actinomycete strains by molecular networking [34]. After their dereplication and subsequent structure elucidation, it was later revealed that these compounds are actually the end product of their polyketide synthase (PKS) assembly line pathway that is, however, also responsible for the production of salinipyrone and pacificanone, linear polyketides that had previously been isolated by a classical approach [35]. Unexpectedly, both appeared to be shunt products of the PKS, as proven by mutagenesis experiments in the rosamicin assembly line [33]. Analogously to peptidogenomics, glycogenomics also holds potential for automation, although this has not yet been implemented.

### 2.2. Correlation-Based Approaches on Larger Paired Datasets: Pattern-Based Genome Mining, Metabologenomics

**Figure 2:**
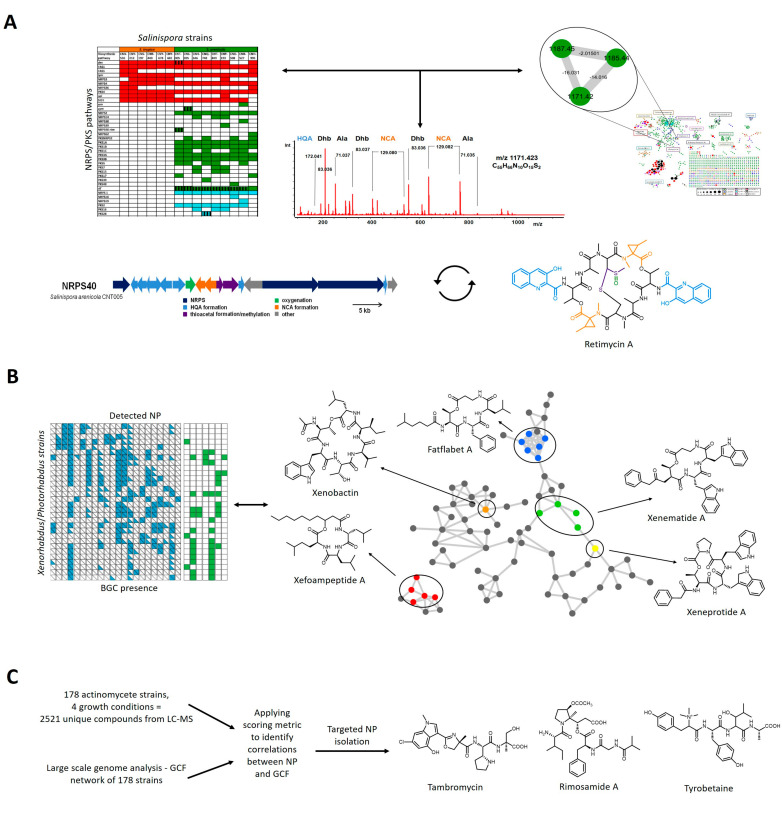
**Discovery examples for correlation-based approaches using paired datasets.** (**A**) Correlation of *Salinispora* strain BGC patterns with a molecular network of 35 strains led to the identification of a candidate peptide, encoded and produced by only one strain in the dataset. The peptide was matched to its NRPS BGC with additional help of the peptidogenomic approach. The structure of the elucidated metabolite retimycin A is depicted. Building blocks are color-coded corresponding to responsible biosynthetic genes [39]. Taken from reference 39 and rearranged with permission of Elsevier. (**B**) *Xenorhabdus* and *Photorhabdus* strains were analyzed for BGC patterns and production of encoded metabolites (left). Subsequent molecular network analysis led to the identification and discovery of several NRPS-derived cyclic depsipeptides (right) [40]. (**C**) Metabologenomic workflow of a 178 strain actinomycetes dataset [41,42,43,44]. An example for the applied scoring metric can be found in reference 21.

Another possibility to link genomic with metabolomic information is the application of correlation-based approaches. Here metabolite patterns, obtained from larger MS datasets of sequenced bacteria, are compared and correlated with their BGC or GCF patterns, derived from comparative analyses of a set of genomes (Figure 2). Notably, these correlations are independent of the chemical class of the detected metabolites. Talented NP producers such as actinomycetes harbor a multitude of BGCs, whereas taxonomically closely related strains characteristically possess overlapping patterns of encoded BGCs. This means that homologous BGCs are frequently encoded in more than one or several related strains, while other BGCs are unique for particular strains. An illustrative model for this phenomenon is the marine actinomycete genus *Salinispora* [36]. 

*Salinispora* species and strains are very closely related on 16S-RNA level, but can be discriminated by the presence of species- or strain-specific patterns of encoded NP BGCs (compare Figure 2A, left). In a remarkable genome mining study led by Ziemert and Jensen, 75 *Salinispora* strains were analyzed and compared regarding their PKS and NRPS pathway variety and evolution [37]. This comprehensive analysis was later extended to 119 strains and to other pathway types such as terpenes and RiPPs [38].

In a pioneering study from the Jensen and Moore labs, the metabolomes of 35 *Salinispora* strains were visualized with GNPS molecular networking and then compared with the NRPS/PKS BGC patterns of the respective strains to establish compound-BGC links [39]. These correlative analyses enabled the linkage of an orphan BGC to the polyketide arenicolide and the targeted isolation, structure elucidation and biological characterization of the cytotoxic, echinomycin-like nonribosomal depsipeptide retimycin A, that was encoded and produced by only one strain in the collection, *S. arenicola* CNT005 (Figure 2A).

An analogous study was performed in the bacterial genera *Photorhabdus* and *Xenorhabdus*, both associated with insects and known for prolific natural product production, by the Bode group [40]. Here, a metabolomic network from HPLC-MS/MS data of 30 strains was created, annotated and compared with BGC patterns in the respective strains (Figure 2B). This study revealed the robust expression of known metabolites under laboratory conditions in a number of strains, but also led to the detection of previously unidentified metabolite classes in these bacteria, such as the novel xefoampeptides and tilivallin and connection to their BGCs. Furthermore, novel depsipeptides named fatflabets and xeneprotides were discovered from analysis of the molecular network, and their structures elucidated. However, a complete BGC for these novel compound families could not be assigned with certainty.

A similar concept to bridge metabolomic and genomic information, termed metabologenomics, was developed in the Metcalf and Kelleher labs (Figure 2C). This approach relies on the establishment of correlations between MS spectra and GCFs in a huge dataset of 830 sequenced actinomycete bacteria, of which 178 were subjected to detailed HPLC-MS metabolic profiling in different culture media [41]. Here, a correlation score between GCF and MS1 data was generated and then applied by searching for exact masses of predicted metabolites in the dataset. Subsequent mining of this extensive, paired dataset for detected metabolites encoded by biochemically interesting BGCs enabled the discovery and characterization of several natural products such as tambromycin [42], rimosamides [43] and tyrobetaines [44] and the detailed investigation of their biosynthetic pathways. 

Zdouc, Sosio and colleagues recently performed a detailed metabolomic investigation of the actinobacterial genus *Planomonospora* [45]. Four of the 72 investigated strains were also genome-sequenced, which allowed for a paired omics analysis leading to the annotation of a BGC for the thiopeptide siomycin and congeners. Furthermore, two novel biaryl-linked tripeptides were isolated after network analysis and their structures elucidated. They represent the first members of a widespread novel class of small RiPPs, encoded by the smallest gene ever reported, as revealed by peptidogenomics and heterologous expression [46]. Metabologenomics was also used by the Duncan lab for the evaluation of a dataset of 25 polar actinomycetes, published in this Special Issue [47]. Their metabolomes were analyzed and correlated to genome data by using a newly developed tool, NP-linker, designed for the automated establishment of NP-BGC correlations [48]. 

In a recent study on the biosynthetic and metabolic diversity in the actinomycete genus *Nocardia*, metabolite-BGC correlations were analyzed based on a double-network approach by the Ziemert and Kaysser groups [49]: A metabolomic network was constructed with GNPS molecular networking [9], as well as a BGC network of all selected strains created via BiG-SCAPE [5]. Then, both networks were analyzed and compared for correlations of molecular families and gene cluster families over the same number of strains. This strategy was validated by the strain-specific discovery and annotation of a battery of unprecedented nocobactin-like siderophores. 

The generation of standardized community repositories such as MIBiG for characterized NP BGCs [50], and the GNPS database for MS/MS data of NP datasets and compounds [9], has improved and facilitated many natural products workflows. However, genome-metabolome links have not been systematically documented and are cumbersome to search for. To overcome this obstacle and to standardize NP-BGC links that can be reused by the community for further projects, recently the paired omics database has been developed and launched [51]. This platform gathers a large number of paired datasets generated by the NP community including links to MS/MS datasets on GNPS and sequence data of characterized BGCs on MIBiG and standardized metabolite-BGC links were generated. This standardized and open community database may be very useful for the application and automation of future correlative network-based approaches and prioritization of novel metabolites and BGCs that are worth investigating. 

An alternative pipeline for bioinformatic analyses of BGCs and compound-cluster matching was developed by the Magarvey lab [52]: Here, a prediction engine (PRISM) identifies and predicts BGC in microbial genomes. A retrobiosynthetic algorithm (GRAPE) performs retrobiosynthetic analyses of known natural products and suggests a likely BGC for these metabolites. A matching algorithm (GARLIC) then compares PRISM and GRAPE outputs and gives matching scores. By that procedure, BGCs with unknown products can be identified with high confidence. Additionally, a “genomes to natural products” (GNP) algorithm matches LC-MS/MS data to BGCs by structure prediction, substructure analysis and in silico fragmentation prediction generating confidence scores of the NP-BGC matches [53]. Notably, this pipeline is restricted to modular PKS and NRPS pathways.

To conclude, in the last decade, several novel MS-guided genome mining workflows and global, standardized mass spectral and BGC databases have been developed and led to a significant number of natural product discoveries. For reliable NP-BGC linkages, these paired omics workflows rely on high quality MS/MS and sequence data and bioinformatic, mass spectral and biosynthetic knowledge. To further expand a paired omics NP mining workflow to other natural product classes, the integrated use of recently developed substructure annotation tools [11], classification-based methods [54] and fragmentation trees [55] together with the use of further improved automated linking approaches [48] are of great promise. Structure elucidation remains a major bottleneck in NP discovery pipelines, and is often limited by the low yields of the NP of interest. However, the development of neural network algorithms for NMR analysis [56] and novel structure elucidation methods such as MicroED [57] may, integrated into the described workflows, further brighten the future for natural product research and enable many exciting discoveries.
